# Identifying Disease Related Genes by Network Representation and Convolutional Neural Network

**DOI:** 10.3389/fcell.2021.629876

**Published:** 2021-02-22

**Authors:** Bolin Chen, Yourui Han, Xuequn Shang, Shenggui Zhang

**Affiliations:** ^1^School of Computer Science, Northwestern Polytechnical University, Xi'an, China; ^2^School of Mathematics and Statistics, Northwestern Polytechnical University, Xi'an, China; ^3^Xi'an-Budapest Joint Research Center for Combinatorics, Northwestern Polytechnical University, Xi'an, China

**Keywords:** identification of disease-related genes, network representation, machine learning, deep learning, convolutional neural network

## Abstract

The identification of disease related genes plays essential roles in bioinformatics. To achieve this, many powerful machine learning methods have been proposed from various computational aspects, such as biological network analysis, classification, regression, deep learning, *etc*. Among them, deep learning based methods have gained big success in identifying disease related genes in terms of higher accuracy and efficiency. However, these methods rarely handle the following two issues very well, which are (1) the multifunctions of many genes; and (2) the scale-free property of biological networks. To overcome these, we propose a novel network representation method to transfer individual vertices together with their surrounding topological structures into image-like datasets. It takes each node-induced sub-network as a represented candidate, and adds its environmental characteristics to generate a low-dimensional space as its representation. This image-like datasets can be applied directly in a Convolutional Neural Network-based method for identifying cancer-related genes. The numerical experiments show that the proposed method can achieve the AUC value at 0.9256 in a single network and at 0.9452 in multiple networks, which outperforms many existing methods.

## 1. Introduction

With the rapid development of high-throughput biological experiment and the wide application of bioinformatics (Guingab-Cagmat et al., 2013), the identification of genes related to human diseases becomes more and more important in understanding the mechanism of disease pathogenesis. Many biological networks (Raval and Ray, 2013) have been used to identify disease related genes, such as genetic interaction networks (Boucher and Jenna, 2013), protein-protein interaction networks (Seebacher and Gavin, 2011), and gene interaction networks (Robert, 2012), etc. Ramsahai et al. (2017) use gene interaction networks to improve the identification of cancer driver genes. Gevaert et al. (2014) use module network interaction of multi-omics data to identify ovarian cancer driver genes.

**Figure 1:**
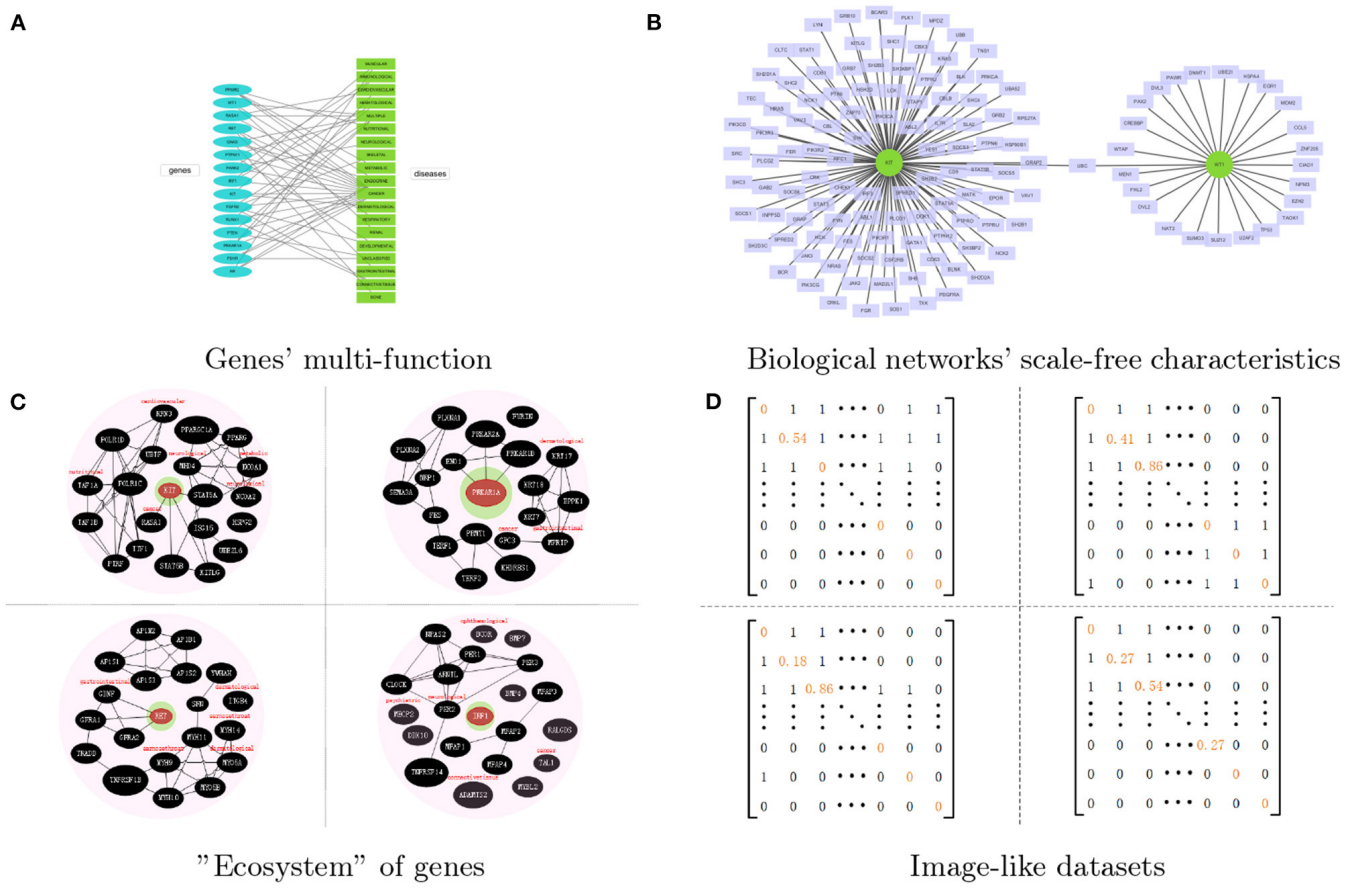
The overall idea of the proposed method. **(A)** The multi-function of genes, which shows genes' multi-tag. **(B)** The biological networks' scale-free characteristics. Two cancer related genes WT1 and KIT are take as the example. **(C)** The four genes' representations processed by our proposed network representation method. Each genes will be surrounded by those selected genes and their tags. All of them come together like an “ecosystem.” **(D)** The image-like datasets which corresponded to the representation of genes.

To achieve the identification of disease related genes by using networks data, many powerful machine learning methods have been proposed from various computational aspects, such as decision tree (She et al., 2010), support vector machine (Choi et al., 2011) and naive Bayes (Yousef et al., 2007). Meanwhile, deep learning methods have also gained big success in identifying disease related genes according to their higher calculation accuracy and efficiency. However, most deep learning methods lack the consideration of multifunction properties of many genes and scale-free characteristics of biological networks, thus resulting in some limitations in the identification of disease related genes. Genes' multi-function and biological networks' scale-free characteristics are shown in Figures 1A,B.

To be more specific, the disease related genes tend to have multi-functions, that is, there may be several genes co-work together to result in some diseases, or one gene is related to multiple diseases. Many graph neural network based representation methods do not consider the multi-function of nodes and also suffer from the limitation of node's degree, especially for scale-free networks (Zhou et al., 2018). Many biological networks, such as PPI networks, pathway co-occurrence networks, gene co-expression networks, and DNA co-methylation network, etc., are not inappropriate with each other, and they often fail to exploit the power of graph neural network. These networks do not accurately explain the similarity between genes and their feature vectors, i.e., those edges only simply indicate that two genes are related to each other, but do not show their global similarities between neighbors. In addition, many genes have no attribute data associated with a disease, but those known multi-function disease related genes contain more information, which make the dimension difference of resulting feature vectors.

Most biological networks have the scale-free property (Boccaletti et al., 2010), which means hubs get more information from their neighbors, while those non-hub nodes get less information from their neighbors. They aren't equivalent. So we need to give “neighbor” a new definition based on similarity instead of the neighbor defined by the original adjacency relationship, which makes node in the scale-free network get information of roughly the same scale. After the node sequence selection and the neighborhood graph construction, we regard node-induced sub-network as the represented object to have a regularization such that every node can get information of roughly the same scale from node-induced sub-network corresponded with itself.

At the same time, the function of a gene is actually the function of the gene's product, that is, the protein's function of the coding gene and the RNA's function of the non-coding gene (Gamermann et al., 2019). The selective expression of genes means genes express in a certain time and space. All of these cause the multifunction properties of many genes. So we need to give the gene a specific environment information to distinguish the gene functions according to the identification of disease class. For one gene, this specific environment is reflected in two aspects: (1) which disease class the gene in this gene's neighbors belongs to; (2) how do this gene's neighbors affect this gene.

In this study, we take node-induced sub-network as the represented object to have a regularization for solving the limitation of node's degree, and add neighbor's environmental characteristics of nodes for solving multifunction of genes, shown in Figure 1C. Then we find a low-dimensional network space for a network, to transfer topological networks into image-like datasets, which can be applied directly by convolutional neural network for identifying cancer-related genes, shown in Figure 1D.

## 2. Materials and Methods

### 2.1. Data Sources

Seven biological networks are employed in this study, which includes four PPI networks, one pathway co-occurrence network, one gene co-expression network, and one DNA methylation dataset. The PPI network are collected from HPRD (Release 9), BioGrid (3.4.143), IntAct (4.2.3.2), and InWeb_IM (2016_09_12). The first three of them are binary PPIs, while the last one is weighted PPIs. The pathway dataset is download from MSigDB (c2.all.v5.2). The expression profiles are obtained from ArrayExpress (E-TABM-305). The DNA methylation dataset is collected from GEO (GSE36064). In this study, we selected those node entries which appear in at least six datasets and resulted in 9189 identical vertices by blurring the differences between proteins and genes (Chen et al., 2017).

The known gene-disease associations are obtained from Goh's paper (Goh et al., 2007) and the OMIM dataset, where 1285 genes are overlapped with the previous 9189 genes. There are 22 classes of diseases, such as cancer, bone, earnosethroat, hematological, etc. Since only genes related to cancer class exhibit dense connections in the human disease gene network, we will take the cancer class for example in this study, and evaluate our proposed method to identify cancer-related genes.

### 2.2. Network Representation

#### 2.2.1. Low-Dimensional Network Space

A graph *G* = < *V, E* > is commonly used to represent a network, where *V* is the vertex set, and *E* is the edge set (Cohen and Havlin, 2010). The space of the adjacency matrix *A* of *G* is called an *n*-dimensional network space, where *n* is the number of nodes. The network representation aims to learn a low-dimensional vector space for a network, in contrast with the *n*-dimensional space (Cui et al., 2017). Obviously, we need to choose an *m*-dimensional network space be the low-rank space, where *m* < < *n*. That is, for every node, we need to choose *m* − 1 nodes as its neighbors, and add environmental characteristics through the relevant information of its neighbors.

#### 2.2.2. Embedding

**Figure 2:**
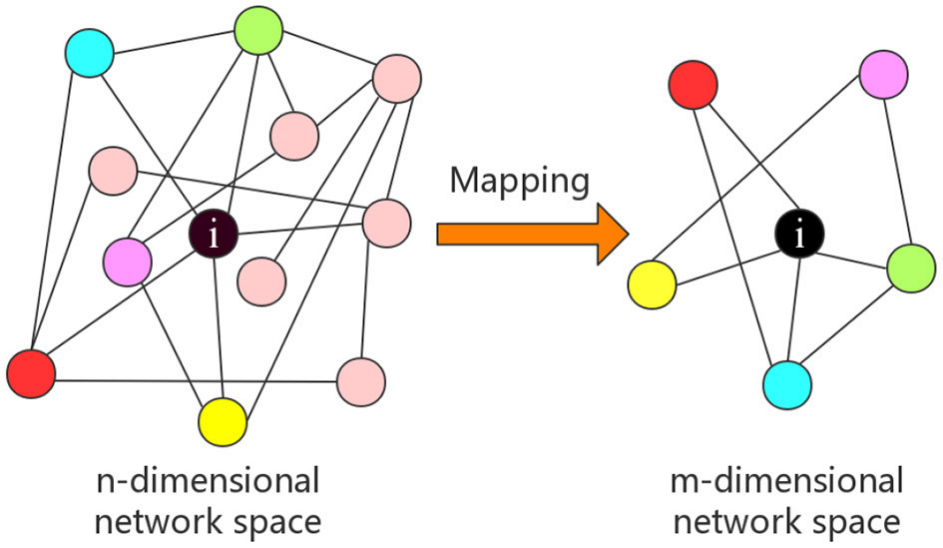
An example of a node's representation, where n-dimensional network space is reduced to m-dimensional network space.

A one-to-one mapping Γ from *n*-dimensional network space to *m*-dimensional network space is established, which is illustrated in Figure 2. A sub-network with *m* nodes is obtained after embedding nodes into the *m*-dimensional graph space as follows.

Firstly, we do the node sequence selection by similarity. We take the row vector as the vector representation of a vertex, e.g., *a*_*i*_ = (*a*_*i*,1_, *a*_*i*,2_, ⋯ , *a*_*i,n*_) is a row vector as the vector representation of the node *v*_*i*_. Define the similarity *S*_*i,j*_ between the node *v*_*i*_ and the node *v*_*j*_ as

(1) $$\begin{array}{l} \;S_{i,j}=\frac{1}{\sum_{k=1}^{n}{\left(a_{i,k}-a_{j,k}\right)}^{2}}. \end{array}$$

The larger *S*_*i,j*_, the more consistent that node *v*_*i*_ and node *v*_*j*_ influence on other vertices, i.e.,the node *v*_*i*_ is similar to the node *v*_*j*_ in this network, which means they may have the similar biological function or take part in similar cellular processes.

Then we rearrange the genes to facilitate the selection of *m* − 1 neighbors. An agglomerative hierarchical clustering algorithm is employed to cluster vertices in the network, where a sequence of leaf vertices $\left({v_{1}}^{\prime },{v_{2}}^{\prime },\cdots \;,{v_{n}}^{\prime }\right)$ is obtained corresponding to the clustering tree. Vertices with higher similarity are closer, while those with less similarity are far away from each other.

Secondly, we do the neighborhood network construction. Given a vertex ${v_{i}}^{\prime }$, a 2*k* + 1 neighborhood field can be obtained by taking ${v_{i}}^{\prime }$ as the center and a receptive field with a radius of *k*, where *m* < *k* < *n*. After this, *m* − 1 vertices can be selected according to their similarity to the center as follows

(2) $$\max\left(\sum_{j=1}^{m-1}S_{v_{i}^{\prime },v_{j}^{\prime }}\right)$$

**Figure 3:**
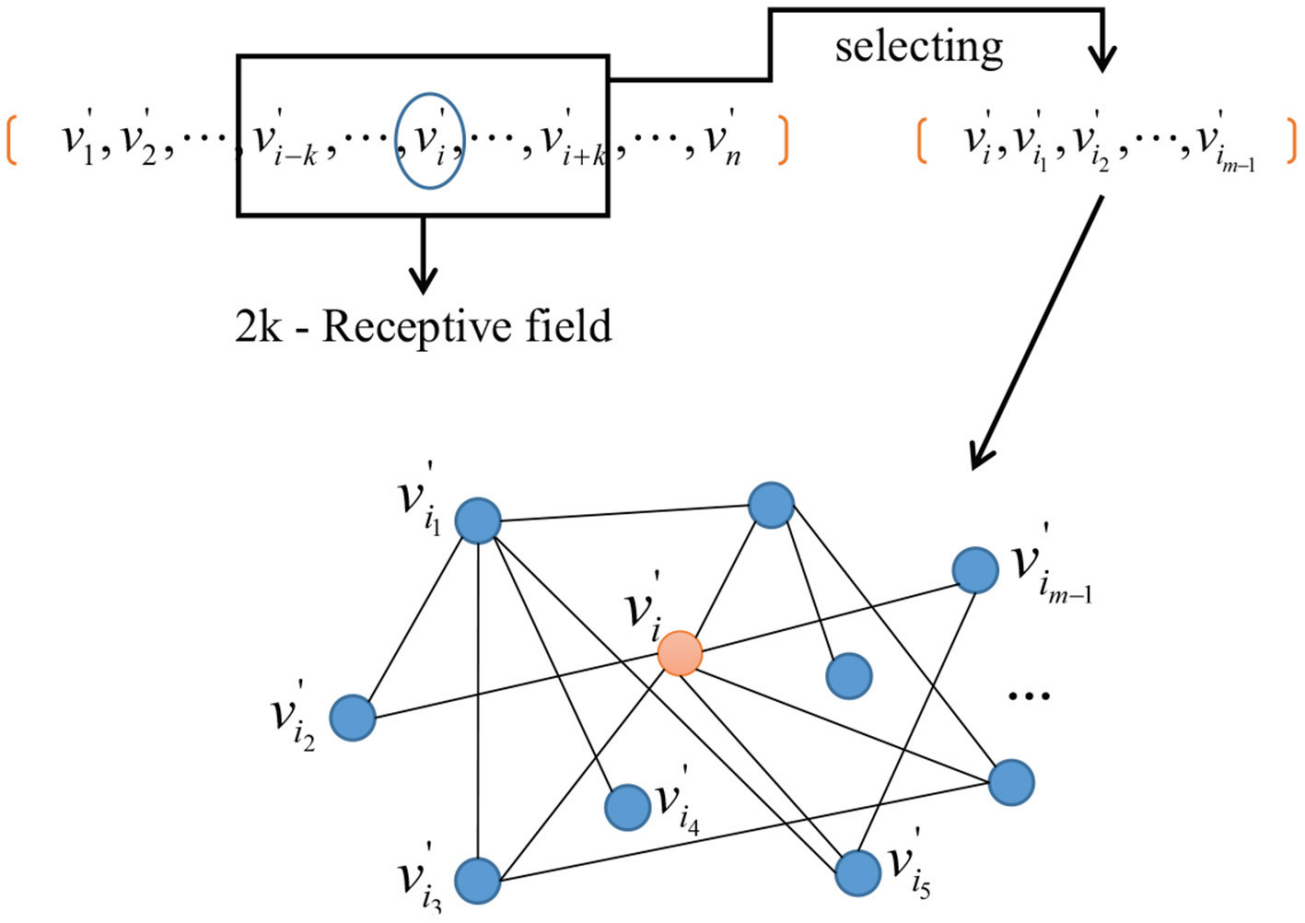
Embedding nodes in m-dimensional network space.

Thirdly, we do the network normalization. Those vertices selected can be embedded to a m-dimensional network space and a sub-network with *m* vertices is obtained as the representation of ${v_{i}}^{\prime }$. The diagram is shown in Figure 3.

#### 2.2.3. Transferring

The adjacent matrix can then be rearranged according to this leaf sequence. By doing this, an *m* * *m* sub-adjacent matrix $A_{{v_{i}}^{\prime }}$ of the above sub-network of vertex ${v_{i}}^{\prime }$ can be obtained. The sub-adjacent matrix $A_{{v_{i}}^{\prime }}$ fully preserves surrounding topological structures of vertex ${v_{i}}^{\prime }$, which reflects how the surrounding vertices affect vertex ${v_{i}}^{\prime }$.

(3) $$A_{v_{i}^{\prime }}=\left[\begin{array}{ccccc} 0 & w_{i,i_{1}} & \cdots & w_{i,i_{m-2}} & w_{i,i_{m-1}}\\ w_{i_{1},i} & 0 & \cdots & w_{i_{1},i_{m-2}} & w_{i_{1},i_{m-1}}\\ \vdots & \vdots & \ddots & \vdots & \vdots \\ w_{i_{m-2},i} & w_{i_{m-2},i_{1}} & \cdots & 0 & w_{i_{m-2},i_{m-1}}\\ w_{i_{m-1},i} & w_{i_{m-1},i_{1}} & \cdots & w_{i_{m-1},i_{m-2}} & 0 \end{array}\right]$$

Moreover, each vertex may also belong to a disease class according to the gene-disease association. We choose neighbors' disease class information of vertex ${v_{i}}^{\prime }$ as its environmental characteristics. A classification matrix $C_{{v_{i}}^{\prime }}$ can also be generated by taking the disease class information as the diagonal element for its *m* − 1 neighbors in the sub-network.

(4) $$C_{v_{i}^{\prime }}=\left[\begin{array}{ccccc} 0 & 0 & \cdots & 0 & 0\\ 0 & c_{i_{1}} & \cdots & 0 & 0\\ \vdots & \vdots & \ddots & \vdots & \vdots \\ 0 & 0 & \cdots & c_{i_{m-2}} & 0\\ 0 & 0 & \cdots & 0 & c_{i_{m-1}} \end{array}\right]$$

Considering the multi-tag caused by the genes' multi-function, we need to process neighbors' disease class information of vertex ${v_{i}}^{\prime }$. For the identified disease class *t*_*id*_ and vertex ${v_{i}}^{\prime }$, whose tag set is $T\left({v_{i}}^{\prime }\right)=\left\{t_{1},t_{2},\cdots \;,t_{j}\right\}$, first we define the tag *t*_*center*_ of the vertex ${v_{i}}^{\prime }$ as follows:

(5) tcenter={tj,if tid∉T(vi′) and d(tj,tid) is minimum;tid,if tid∈T(vi′);0,if T(vi′)=o.

Second, for the tag *t*_*center*_ of the vertex ${v_{i}}^{\prime }$ and vertex ${v_{i_{k}}}^{\prime }$ whose tag set is $T\left({v_{i_{k}}}^{\prime }\right)=\left\{t_{1},t_{2},\cdots \;,t_{j}\right\}$, we define *c*_*i*_*k*__ the as follows:

(6) cik={tj/22,if tcenter∉T(vik′) and d(tj,tcenter) is minimum;tcenter/22,if tcenter∈T(vik′);0,if T(vik′)=o.

where *d*(*t*_*i*_, *t*_*j*_) means the centroid linkage of *t*_*i*_ and *t*_*j*_.

Then an image-like two-dimensional matrix $E_{{v_{i}}^{\prime }}$ can be obtained by adding $A_{{v_{i}}^{\prime }}$ and $C_{{v_{i}}^{\prime }}$ together. This image-like two-dimensional matrix of the sub-network is a network representation of vertex ${v_{i}}^{\prime }$, which preserves rich structural information from sub-network's weighted adjacency matrix and the important network properties from classifications' matrix.

(7) $$E_{v_{i}^{\prime }}=A_{v_{i}^{\prime }}+C_{v_{i}^{\prime }}=\left[\begin{array}{ccccc} 0 & w_{i,i_{1}} & \cdots & w_{i,i_{m-2}} & w_{i,i_{m-1}}\\ w_{i_{1},i} & c_{i_{1}} & \cdots & w_{i_{1},i_{m-2}} & w_{i_{1},i_{m-1}}\\ \vdots & \vdots & \ddots & \vdots & \vdots \\ w_{i_{m-2},i} & w_{i_{m-2},i_{1}} & \cdots & c_{i_{m-2}} & w_{i_{m-2},i_{m-1}}\\ w_{i_{m-1},i} & w_{i_{m-1},i_{1}} & \cdots & w_{i_{m-1},i_{m-2}} & c_{i_{m-1}} \end{array}\right]$$

## 3. Experiments and Results

### 3.1. The Comparison of Traditional Machine Learning Methods and Convolutional Neural Network

Considering the single weighted PPI network (InWeb_IM), we do a binary classification of cancer-related genes as an example. There are a total of 1,285 genes related to 22 classifications of diseases known, of which 178 cancer-related genes are positive samples, and the same number of negative samples are randomly selected from other diseases' classification.

Since there are 22 classifications of diseases, one of which is unclassified, so we choose 21-dimensional network space as *m*-dimensional network space be the low-rank space. After the processing of the network representation method, we embed genes of the network to the 21-dimensional network space, then we transfer those 21-dimensional sub-networks to image-like 21*21 matrix.

**Figure 4:**
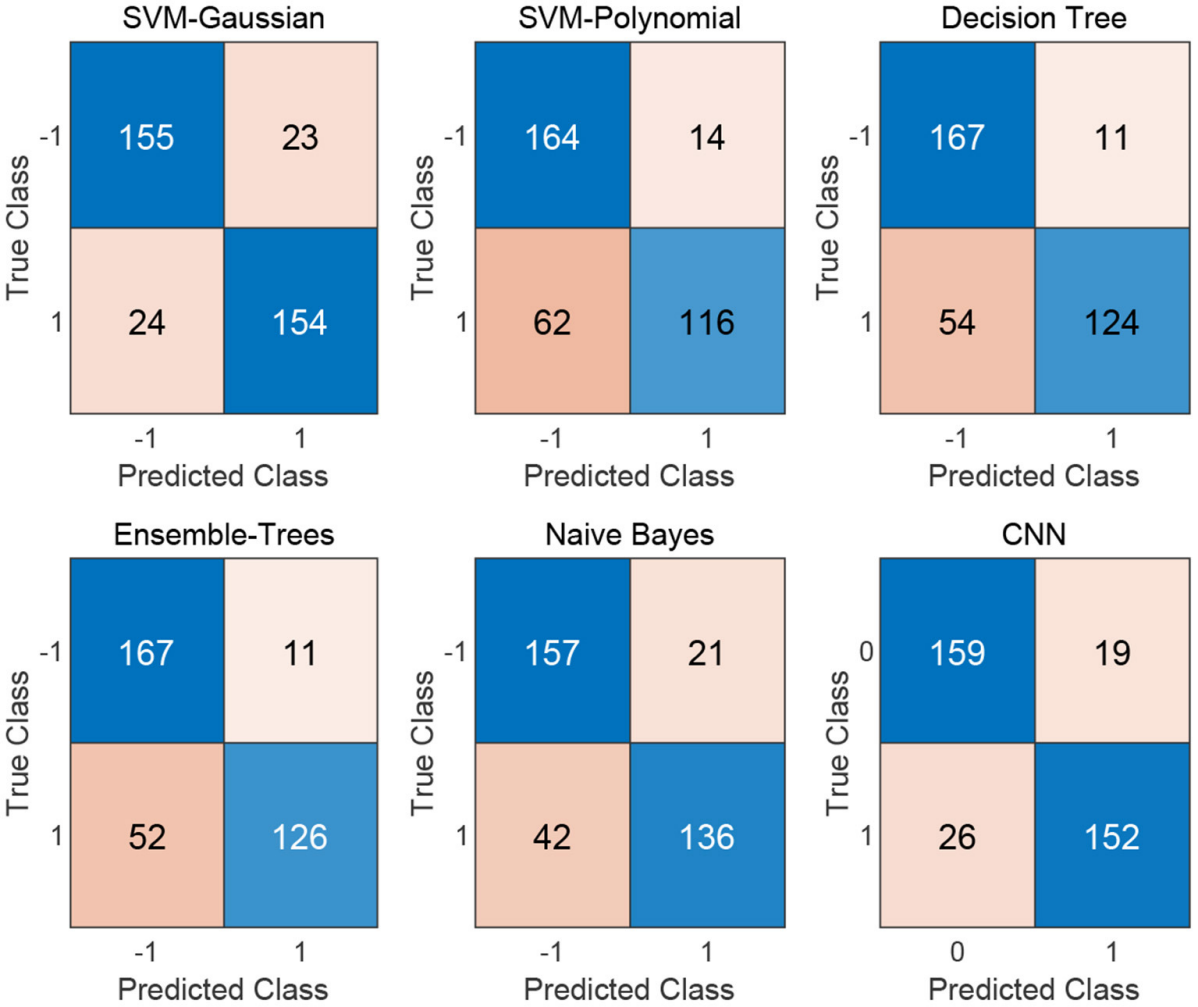
Confusion matrix of the five traditional machine learning methods and the Convolutional Neural Network method.

**Figure 5:**
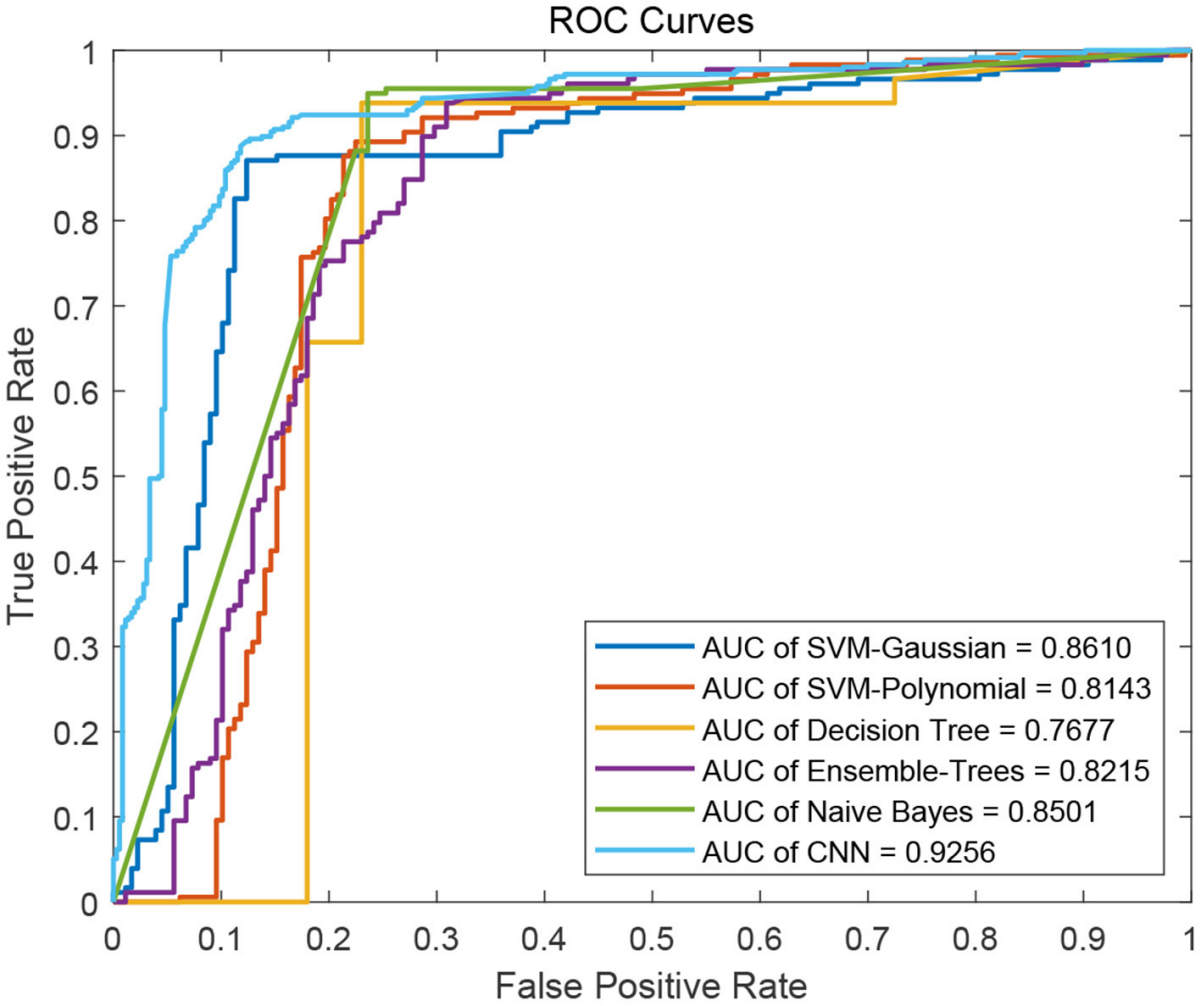
ROC curves of the five traditional machine learning methods and the Convolutional Neural Network method.

We flat the image-like 21*21-dimensional matrix as a 441-dimensional vector, and do binary classification of cancer-related genes using traditional machine learning methods, such as Decision Trees, Support Vector Machines and Naive Bayes. At the same time, we use image-like 21*21-dimensional matrix directly to do binary classification of cancer-related genes by Convolutional Neural Network. Results are shown in Figures 4, 5.

For traditional machine learning methods, although the flattening operation breaks the physical meaning of the original image-like matrix, other machine learning methods have good performance, in addition to DT. Among the five machine learning methods, the best performance in the binary classification of cancer-related genes is the Gaussian kernel Support Vector Machine [SVM(Gau)] method, with the precision 0.8701, recall 0.8651, F1-measure 0.8676, and the AUC value of 0.8610. From the classification results, the other four machine learning methods all focus on the identification of negative samples, except for the SVM(Gau) method. The SVM(Gau) method has roughly the same ability of the identification for positive and negative samples.

For Convolutional Neural Network, its Performance is better than those five traditional machine learning methods according to the f1-measure and the AUC values. From the classification results, this classifier has roughly the same ability of the identification for positive and negative samples, without focusing on one of them.

Table 1 shows the results of comparing the Convolutional Neural Network method with five traditional machine learning methods.

**Table 1:** A summary of five machine learning algorithms and CNN.

**Method**	**Accuracy**	**Precision**	**Recall**	**f1-measure**	**AUC**
SVM(Gua)	0.8679	0.8701	0.8651	0.8676	0.8610
SVM(Pol)	0.7865	0.8923	0.6517	0.7532	0.8143
DT	0.8174	0.9185	0.6966	0.7923	0.7677
ET	0.8230	**0.9197**	0.7079	0.8000	0.8215
NB	0.8230	0.8662	0.7640	0.8119	0.8501
CNN	**0.8736**	0.8539	**0.8889**	**0.8710**	**0.9256**

*The bold values indicate the best prediction accuracy in that column*.

### 3.2. The Comparison of Different Networks by Convolutional Neural Network

**Figure 6:**
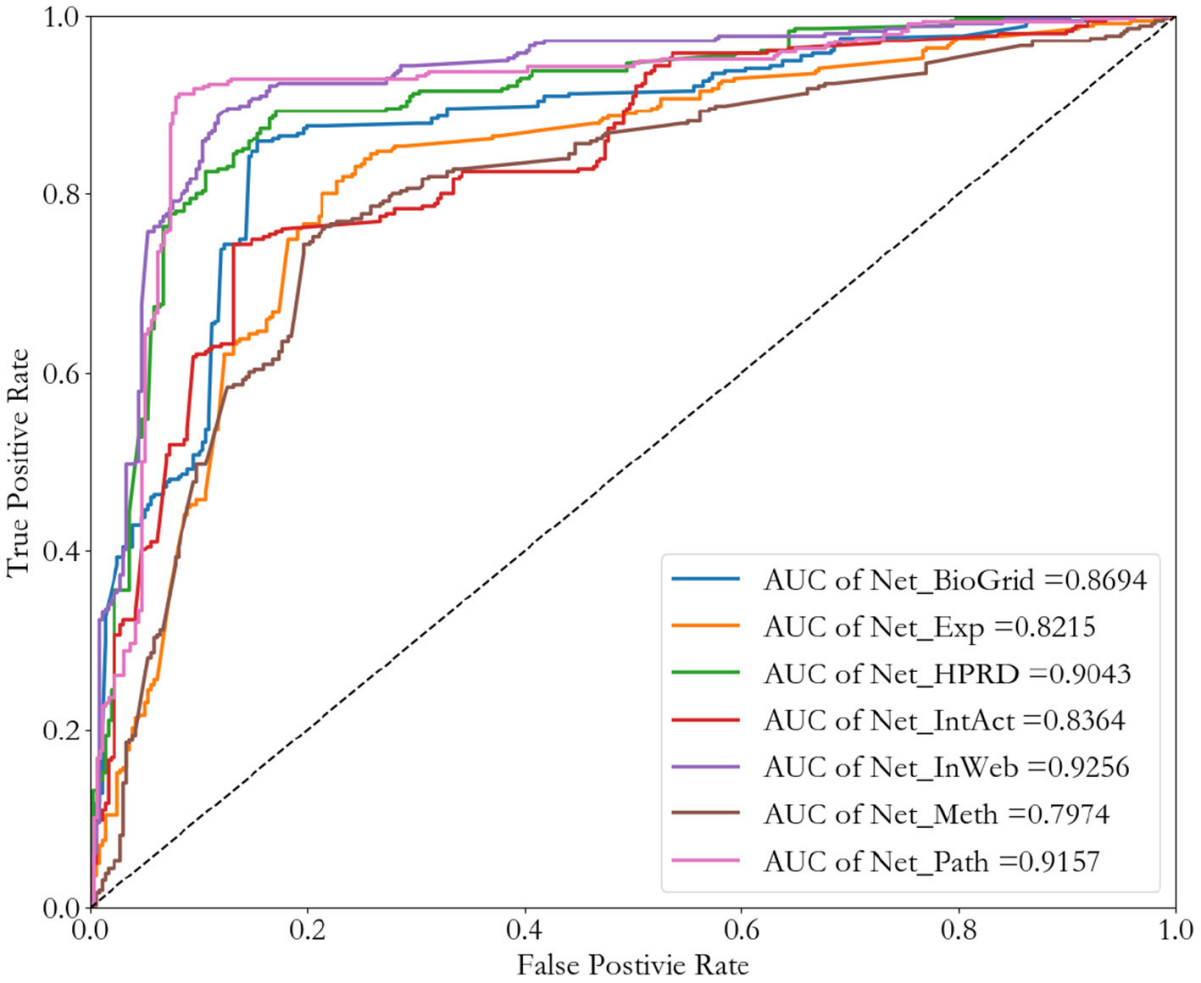
ROC curves of different networks by using Convolutional Neural Network method.

To verify that our network representation method is applicable to different types of networks, we consider all these Seven biological networks, which include four PPI networks, one pathway co-occurrence network, one gene co-expression network, and one DNA methylation dataset. We also do a binary classification of cancer-related genes by Convolutional Neural Network as an example. Results are shown in Figure 6.

Comparing the four PPI networks (NT_HPRD, NT_BioGrid, NT_IntAct, and NT_InWeb), weighted PPI network (NT_InWeb) performs best. This is in line with our expectations, because the weighted PPI network contains more detailed information than binary PPI network. Among all these seven networks, the best performance in the binary classification of cancer-related genes is weighted PPI network (NT_InWeb), and other networks also have a good performance, but the DNA methylation network (NT_meth).

**Figure 7:**
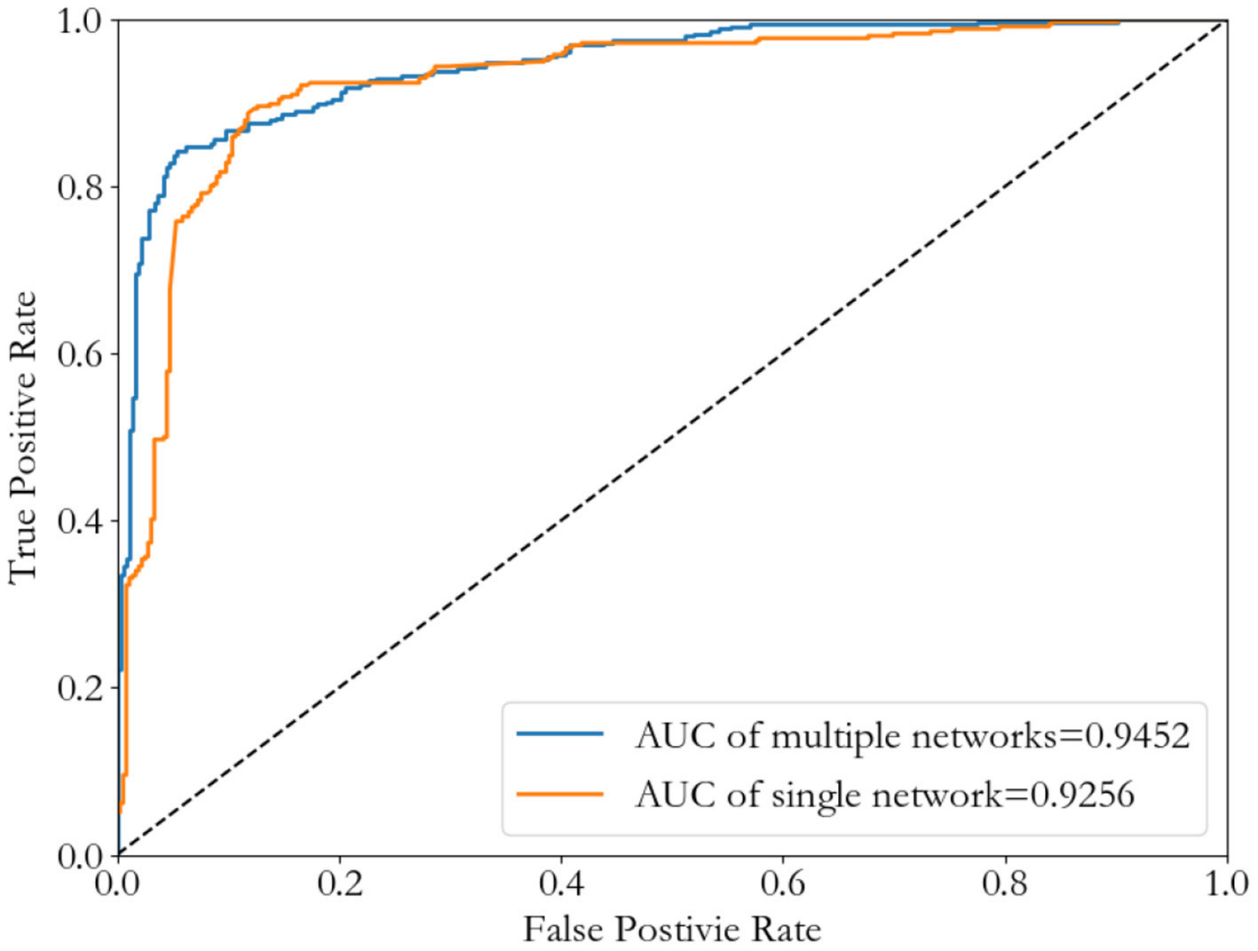
ROC curves of the Convolutional Neural Network method by using either a single network (InWeb_IM PPI) or seven multiple networks.

Through comparison, we find that the network with more information is more conducive to the identification of cancer-related genes. Therefore, we hope to merge the information of the seven networks to achieve the purpose of cancer-related genes' identification. We choose the node-induced sub-network caused by weighted PPI network (NT_InWeb), and add image-like 21*21 matrix evenly, which is corresponding to this node-induced sub-network in other six networks. Then we do a binary classification of cancer-related genes by Convolutional Neural Network to compare with the single weighted PPI network (NT_InWeb). Results are shown in Figure 7.

Although there is little difference in AUC between the two, seven multiple networks is slightly higher than the single network. And the best threshold with the least classification error of seven multiple networks is lower than the single network. Table 2 shows the results of comparing the single network (NT_InWeb) and seven multiple networks.

**Table 2:** A comparison of the single network and seven multiple networks.

**Network**	**ACC**	**PPV**	**TPR**	**TNR**	**f1-measure**
Single network (InWeb_IM)	0.8736	0.8889	**0.8539**	0.8933	0.8710
Seven networks	**0.8904**	**0.9484**	0.8258	**0.9551**	**0.8829**

*The bold values indicate the best prediction accuracy in that column*.

### 3.3. The Comparison With Previous Network Representation Method

**Figure 8:**
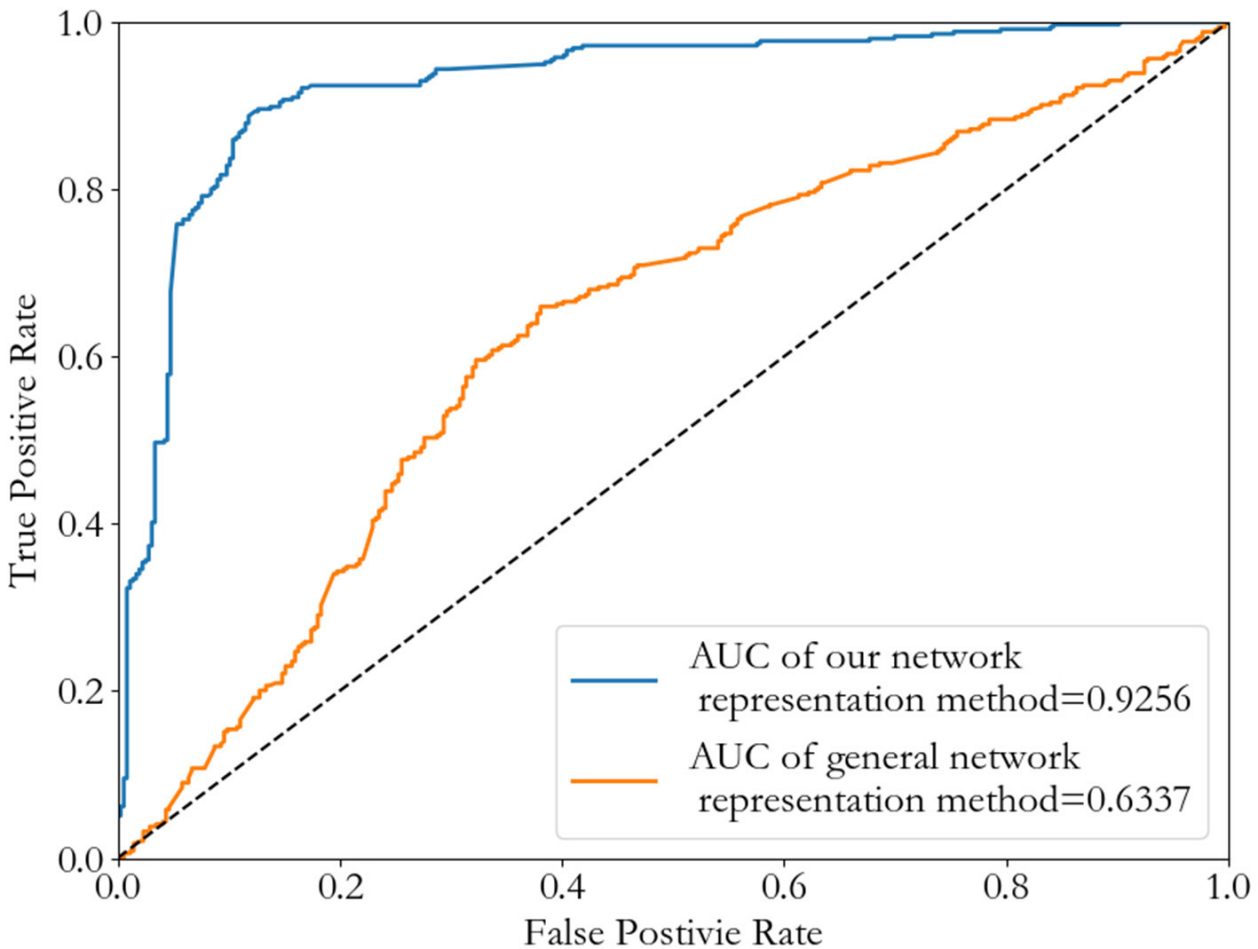
ROC curves of Convolutional Neural Network method by using different network representation methods.

To verify that our network representation method is better than general network representation method without environmental characteristics, we consider the single weighted PPI network (InWeb_IM) to compare the two. After the node sequence selection by similarity, we choose $\left({v_{i}}^{\prime },{v_{i+1}}^{\prime },\cdots \;,{v_{i+20}}^{\prime }\right)$ as the neighborhood of the node ${v_{i}}^{\prime }$, and rearrange the 21*21 adjacent matrix according to this neighborhood sequence as the representation of node ${v_{i}}^{\prime }$. We also do a binary classification of cancer-related genes by Convolutional Neural Network as an example. Results are shown in Figure 8. It's clear to see our novel network representation method achieve a higher AUC value than general network representation method without environmental characteristics, and its best threshold is also lower.

## 4. Conclusions

In this paper, we have proposed a novel network representation method, aiming to find a low-dimensional network space for a network, by transferring topological networks into image-like datasets. It can be applied directly by Convolutional Neural Network. Compared with traditional machine learning methods, Our network representation method can process network data directly for identifying disease related genes by Convolutional Neural Network, and achieve a very high AUC value in the binary classification of cancer-related genes.

## Data Availability Statement

The original contributions presented in the study are included in the article/supplementary material, further inquiries can be directed to the corresponding author/s.

## Author Contributions

BC initialized this study. YH and BC discussed many times to finalized the work plan. XS and SZ gave suggestions many times to modify this study. YH conducted the numerical experiments and drafted the manuscript. All authors read the manuscript and revised it, and agreed with the final version.

## Conflict of Interest

The authors declare that the research was conducted in the absence of any commercial or financial relationships that could be construed as a potential conflict of interest.
